# Sensor Network for Analyzing Upper Body Strategies in Parkinson’s Disease versus Normative Kinematic Patterns

**DOI:** 10.3390/s21113823

**Published:** 2021-05-31

**Authors:** Paola Romano, Sanaz Pournajaf, Marco Ottaviani, Annalisa Gison, Francesco Infarinato, Claudia Mantoni, Maria Francesca De Pandis, Marco Franceschini, Michela Goffredo

**Affiliations:** 1 IRCCS San Raffaele Roma, 00166 Rome, Italy; paola.romano@sanraffaele.it (P.R.); sanaz.pournajaf@sanraffaele.it (S.P.); annalisa.gison@sanraffaele.it (A.G.); francesco.infarinato@sanraffaele.it (F.I.); claudia.mantoni@sanraffaele.it (C.M.); marco.franceschini@sanraffaele.it (M.F.); michela.goffredo@sanraffaele.it (M.G.); 2 San Raffaele Cassino, 03043 Cassino, Italy; maria.depandis@sanraffaele.it; 3 Department of Human Sciences and Promotion of the Quality of Life, San Raffaele University, 00166 Rome, Italy

**Keywords:** upper limb, Parkinson’s disease, Box and Block test, inertial sensors network, biomechanics analysis, kinematic data, hand trajectories

## Abstract

In rehabilitation, the upper limb function is generally assessed using clinical scales and functional motor tests. Although the Box and Block Test (BBT) is commonly used for its simplicity and ease of execution, it does not provide a quantitative measure of movement quality. This study proposes the integration of an ecological Inertial Measurement Units (IMUs) system for analysis of the upper body kinematics during the execution of a targeted version of BBT, by able-bodied persons with subjects with Parkinson’s disease (PD). Joint angle parameters (mean angle and range of execution) and hand trajectory kinematic indices (mean velocity, mean acceleration, and dimensionless jerk) were calculated from the data acquired by a network of seven IMUs. The sensors were applied on the trunk, head, and upper limb in order to characterize the motor strategy used during the execution of BBT. Statistics revealed significant differences (*p* < 0.05) between the two groups, showing compensatory strategies in subjects with PD. The proposed IMU-based targeted BBT protocol allows to assess the upper limb function during manual dexterity tasks and could be used in the future for assessing the efficacy of rehabilitative treatments.

## 1. Introduction

Upper limb impairment can result from a number of different conditions or pathologies, including stroke, Parkinson’s disease, musculoskeletal disorders, infantile cerebral palsy, etc. People who undergo rehabilitation treatments of the upper limb are generally assessed using functional and motor scales [1,2,3,4] in order to characterize the efficacy of a specific therapy or the evolution of the disease over time. The performance related to dexterity, strength, upper limb function, and Activities of Daily Living (ADLs) is typically evaluated via a set of validated clinical tests [5,6].

The recovery of manual dexterity is particularly important because the ability to use the hands in a skillful, coordinated way to grasp and manipulate objects is correlated to a good level of quality of life [7]. One of the most used tests to assess manual dexterity is the Box and Blocks Test (BBT) [8], which has been applied in different pathologies such as stroke [9], multiple sclerosis [2], traumatic brain injuries [2], Parkinson’s disease [10], and upper limb amputation [11]. The test provides an essential measure for upper limb dexterity and motor coordination and consists of moving, one by one, the maximum number of blocks from one compartment of a box to another of equal size within 60 s. The BBT is commonly used in clinical practice because it is a quick, simple, and inexpensive test [8]. Moreover, it is a well-validated timed performance measure of upper-limb function with good reliability [2]. Specifically, in subjects with Parkinson’s disease (PD), the BBT is a good predictor of physical performance in daily living [10]. However, the BBT returns a global score representing the motor task and does not include any assessments of upper limb movement quality. In some cases, in addition to counting the number of cubes moved, clinicians observe a video recorded during the execution of the BBT and qualitatively describe the patient’s motor performance. However, in these circumstances, the clinical analysis is subjective, with low inter-rater reliability, and it is time-consuming. To this extent, the instrumented motion analysis during the BBT would be interesting to integrate the assessment of manual dexterity with the study of upper limb movement quality. Specifically, the kinematic analysis during the BBT could allow obtaining an accurate and objective assessment of the movements of the upper limb and trunk, and thus to find potential compensatory strategies used by the subject to perform the task [12,13]. The literature on the instrumented motion analysis during upper limb clinical tests is wide but heterogeneous in terms of the technology employed for the analysis and of the typology of tasks analyzed [14,15,16,17,18,19,20,21,22,23,24,25,26,27,28]. The most common technologies used for analyzing upper limb kinematics in the clinical setting are stereophotogrammetry and Inertial Measurement Units systems.

The stereophotogrammetry based on reflective markers and optoelectronic sensors has been used in different protocols for the upper limb analysis [15], including modified versions of BBT [16,17]. Specifically, Hebert et al. [16] collected data in 16 able-bodied participants to establish normative kinematics during the BBT. The subjects performed the motor tasks with both arms in standing and seated positions and the results highlighted significant differences between the two conditions in axial trunk rotation, medial-lateral sternum displacement, and anterior-posterior hand displacement. Kontson et al. [17], on the other hand, assessed both upper body kinematics and postural control with an integrated movement analysis framework based on stereophotogrammetry and ground force data. The analysis of 19 able-bodied subjects conducting a modified version of the BBT demonstrated the feasibility of the experimental protocol measure and the average trends of the analyzed population.

Inertial Measurement Units (IMUs) systems have been used as an alternative to stereophotogrammetry because of a series of features that make them easier to use, especially in the clinical setting: ecological environment (outside the movement analysis laboratory), simple application of the sensors (through Velcro strips over the patient’s clothing, unlike the stereophotogrammetry where the reflective markers are applied to the skin), and low costs. For these reasons, the literature includes several studies on the use of IMUs for the kinematic analysis of upper limb movement [19,20,21,22]. However, the IMU-based quantitative evaluation of clinical arm tests is limited to the Action Research Arm Test [23], the Fugl–Meyer, and the Wolf Motor Function Test in post-stroke subjects [24,25] and three items of the Unified Parkinson’s Disease Rating Scale (UPDRS) in subjects with PD [26]. To our best knowledge, only Zhang et al. attempted to automatically assess dexterity with a multimodal wearable sensors-based BBT system [18] based on both electromyography and IMUs. Results from both healthy subjects and people with mild cognitive impairment showed that the multimodal instrumented BBT was feasible and accurate. In this context, although the analysis of upper limb kinematics during a motor task of manual dexterity, such as the BBT, could be particularly relevant in subjects having typical impairment in grasping and manipulating objects, such as PD [13,29], the literature lacks studies on this topic.

This study aims to assess the upper body kinematics during the BBT with an ecological IMU-based system. Specifically, the protocol aims to characterize the movement of the upper body in subjects with PD, comparing them with the data obtained from able-bodied subjects. We hypothesize that the IMU-based BBT would allow us to characterize the quality of the movements and thus quantify the compensatory strategies typical of subjects with PD [13,30,31]. By meeting these objectives, the IMU-based BBT could be a potential system for the standardized assessment of the upper limb.

## 2. Materials and Methods

### 2.1. Study Design

This was an observational single-session-assessment pilot study assessing upper body kinematics during the execution of a BBT motor task, comparing able-bodied persons with subjects with PD. The study was carried out in the neurorehabilitation research laboratory and rehabilitation bioengineering laboratory of IRCCS San Raffaele Roma (Rome, Italy).

### 2.2. Participants

#### 2.2.1. Control Group

Adult able-bodied subjects between 60 and 80 years old without upper limb pathologies (peripheral neurological damage, serious inflammatory degenerative joint diseases, fracture, or trauma results), cognitive and/or severe visual deficit were recruited as the Control Group (CG).

#### 2.2.2. Parkinson’s Disease Group

Individuals with idiopathic PD consecutively referred for counseling and outpatient rehabilitation management were included as PD Group (PDG) if they meet the following inclusion criteria: diagnosis of idiopathic PD by UK Brain Bank criteria; Hoehn and Yahr-H&Y stage 2–3; aged between 50 and 80 years old; able to maintain a sitting position on a chair without support for at least 30 min (Trunk Control Test, TCT ≥ 48) [32]; moderate disease-related upper limb motor performance deficit (i.e., Unified Parkinson’s Disease Rating Scale, UPDRS Part II, items 8, 9, 10, 11, 12, 16 = 2–3; Part III, items 20, 21, 22, 23, 24, 25 = 2–3); stable symptomatic medications during the month before enrollment; and provided written informed consent. We excluded individuals with left-side motor symptom predominance; inability to understand study instructions (Informed Consent Test of Comprehension); cognitive impairment (Montreal Cognitive Impairment Assessment, MoCA < 26 [33]); severe visual deficit; alcohol or drug abuse (including dopamine dysregulation syndrome); active depression; anxiety or psychosis interfering with the use of the equipment or testing; coexisting disabling neurological or orthopedic disorders at upper limb; and previous brain surgery (including pallidotomy, thalamotomy, or deep brain stimulation).

### 2.3. Clinical Assessments

Overall disease-related disability was assessed by the total UPDRS and subtotal UPDRS part II and III scores [34] the trunk stability by TCT, and gross manual dexterity by standard BBT of both dominant and non-dominant sides [5]. All clinical measures were collected in the “ON medication” phase (i.e., 1 h after oral consumption of the usual Levodopa dose and always in the morning to minimize variability). The assessments were by trained professionals. The UPDRS was scored by clinicians specialized in movement disorders and trained for its administration and interpretation.

### 2.4. Experimental Setup

**Figure 1:**
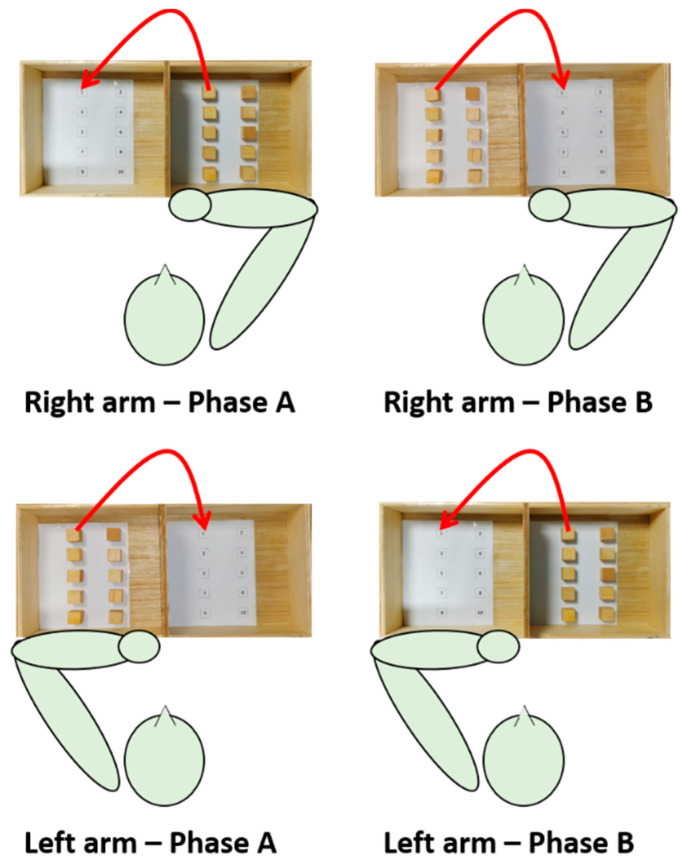
Experimental setup of the IMU-based targeted BBT.

The study took place at the laboratories of IRCCS San Raffaele Roma equipped with the IMU sensors network MOVIT (Captiks srl, Rome, Italy). The experimental setup is shown in Figure 1.

The subject was seated on a stable chair (without backrest and armrests) adjustable in height so that hip and knee angles equal to 90° are formed. A height-adjustable table was placed in front of the subject; the heights of the table and seat were adjusted so that the subject formed 90-degree elbow angles by resting the forearms on the table. A standardized BBT box (53.7 × 25.4 × 8.5 cm^3^) was placed on the table so that the 15.2 cm high division was in correspondence with the median-lateral axis of the subject and at a distance such that the subject reached the vertex of the box with the distal point of the metacarpal bone of the middle finger.

After the IMU sensors network calibration phase, seven IMUs were applied using elastic bands fastened with Velcro strip on the following anatomical points: front head; C5; T10; L5; mid arm; mid forearm; and hand (III° metacarpus). Data were collected at a rate of 60 Hz. A digital video camera was also incorporated into the system to capture frontal recordings of the subjects performing each task. After the measurement of anthropometric data (i.e., distances between the following: spinous processes of C5–T10; T10–L5; acromion processes; acromion process–olecranon process; olecranon process–styloid process), the participants were asked to execute the motor task with both the dominant and the non-dominant arm at self-paced velocity.

The motor task was a modified version of the BBT (namely targeted BBT) and consisted of transporting each block over the partition starting with the innermost left block (n° 1), and moving across the rows following the numbering, and placing it in the corresponding position as accurately as possible. Each IMU-based targeted BBT task was composed of two phases, phase A (ipsilateral subtask) and phase B (contralateral subtask). Each task was executed twice with each arm; since the first execution allowed the subject to become familiarized with the experiments, data analysis was conducted on the second execution only.

### 2.5. Data Processing and Statistical Analysis

The Captiks Motion Analyzer software returned the joint angles curves in the sagittal, frontal, transverse planes, and the calibrated quaternions. The following angles were analyzed in the study: wrist Flexion-Extension (F-E); Ulnar Radial Deviation (URD); forearm Prone-Supination (P-S); elbow F-E; shoulder F-E; shoulder Abduction-Adduction (A-A); shoulder Rotation (R); trunk F-E; and trunk R. The data were segmented into ten trials, where the trial start was defined as the initiation of the approach to pick up a block, and the trial end was defined as the release of the block. The angles were analyzed by with an in-house software developed in MATLAB R2020a (The MathWorks, Natick MA, USA). The following joint angle parameters were calculated for each trial and for each subject: the mean angle and the Range of Execution (ROE). The ROE was defined as the difference between the maximum and the minimum values of each joint angle during the motor task. Moreover, the mean temporal trends of each joint angle were plotted with respect to the trial completion percentage.

The 3D hand trajectory was estimated by using calibrated quaternions and anthropometric data. The first objective was to obtain the spatial orientation of each body segment with respect to the absolute reference system acquired during the calibration phase; following software specifications, the calibrated quaternion coefficients allowed to derive the elements of rotation matrix (*R*) of the reference system of each device integral with a body segment with respect to the absolute reference system. Secondly, vector coordinates $\vec{v}$ of distance between consecutive sensors were derived from the anthropometric data. The transformation matrices between two consecutive coordinated systems of each proximal and distal segment couples were obtained by placing column$\vec{v}$ into rotation matrices multiplication, as detailed in the following formula:

Tdistalproximal=(Rproximal*Rdistalv→0001)

Considering the pelvis as a motionless segment during the task, the pose of the hand relative to the pelvis was obtained by concatenating the transformation matrices connecting distal and proximal segments, in accordance with the following formula [35]:

Thandpelvis=TT10pelvis∗TC7T10∗TarmC7∗Tforearmarm∗Thandforearm

The hand trajectory was achieved by selecting the x, y and z axis coordinates from the resulting transformation matrix and the following parameters were calculated: mean velocity (V_m_); mean acceleration (A_m_); and DimensionLess Jerk index (DLJ). The DLJ is a measure of the movement smoothness, i.e., as an assessment of the quality of the gesture related to its continuity and interruptions absence [36,37]. In this study, we calculated the DLJ index to estimate the shape of trajectory, considered as the most effective and common smoothness measure [38]. It is defined as follows:

$$\mathrm{DLJ}=-\frac{{\left(t_{2}-t_{1}\right)}^{5}}{v_{peak}^{2}}{\int_{t_{1}}^{t_{2}}\left|\frac{d^{2}v(t)}{dt^{2}}\right|}^{2}dt,$$

where *t*_1_ and *t*_2_ are the instants of gesture start and end respectively, *v*(*t*) is the movement speed and *v_peak_* is its maximum in the interval [*t*_1_, *t*_2_]. Values of DLJ closer to 0 correspond to a smoother movement shape.

All estimated parameters were averaged within-subject among blocks and then statistical analysis was conducted. Since data were non-normally distributed (Shapiro-Wilk test), the Mann–Whitney test between CG and PDG for each parameter was applied with a significance level set to *p* < 0.05 (IBM SPSS Statistics for Windows, Version 26.0. Armonk, NY, USA: IBM Corp).

### 2.6. Ethical Aspects

This study was conducted in accordance with the Declaration of Helsinki and was approved by the local ethics committee (no. PR 19/34 of December 2019). Participants were included in the study after signing informed consent.

## 3. Results

Thirteen subjects with PD (in the PDG) and eleven able-bodied subjects (in the CG) were enrolled in the study. Two patients in the PDG were excluded from the analysis because of the presence of artifacts in the IMU data. Table 1 describes the clinical and demographic characteristics of the participants included in the study.

All participants conducted the IMU-based targeted BBT tasks without any difficulties. The data analysis calculated the joint angle parameters shown in Table 2.

The mean joint angles registered significant differences between the PDG and the CG in the following angles: wrist F-E (both arms, both phases) and URD (both arms, both phases); forearm P-S (both arms, both phases); shoulder F-E (non-dominant arm, both phases) and R (both arms, both phases); trunk F-E (non-dominant arm, both phases) and R (both arms, both phases).

The ROE index exhibits statistically significant inter-group differences in the following angles: wrist F-E (both arms, both phases) and URD (dominant arm phase B; non-dominant arm both phases); forearm P-S (dominant arm phase A); shoulder F-E (both arms, both phases), A-A (both arms, both phase) and R (both arms, both phases); trunk F-E (dominant arm both phases; non-dominant arm phase A) and R (non-dominant arm phase A).

**Figure 2:**
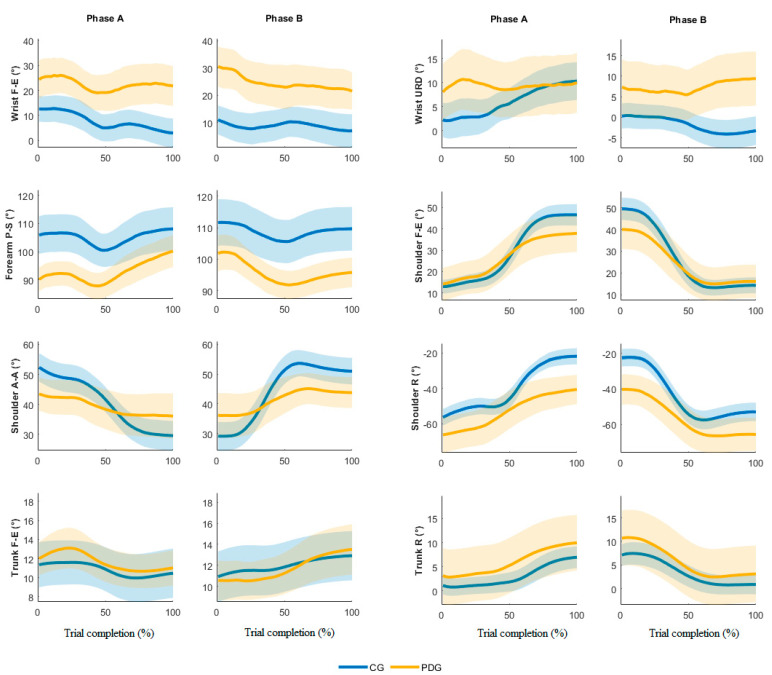
Time-normalized angle joints in phases A and B, dominant side.

Figure 2 depicts the mean joint angle trajectories (dominant arm) over the trial complexion % for both phase A and B (the highlighted line and the shaded color represent the averaged trajectory among blocks and subjects and its standard error, respectively). The analysis of the angle trends revealed the proposed IMU-based targeted BBT protocol is able to detect different motor strategies employed during the movement execution. Specifically, the kinematics of the PDG is characterized by a limited range of movement of the shoulder and a compensatory strategy of the trunk.

**Figure 3:**
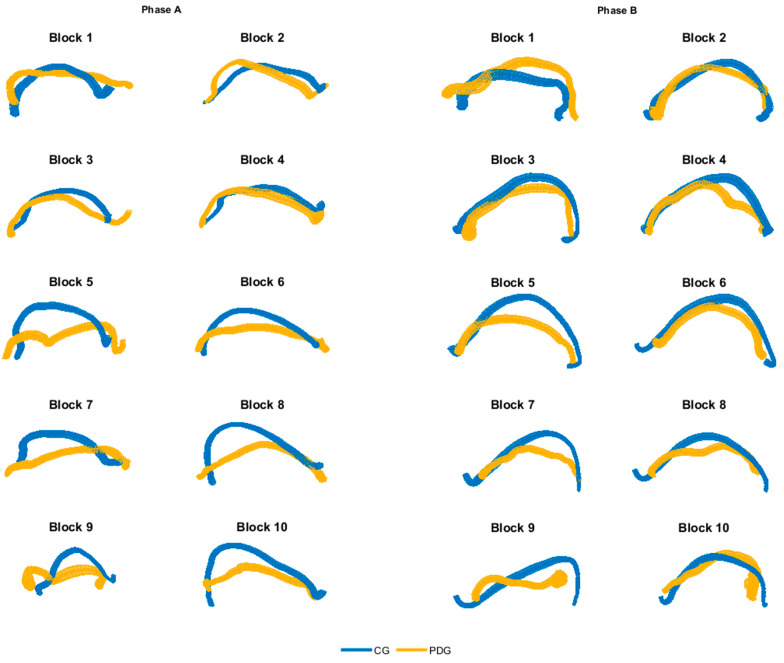
Averaged hand trajectories for both CG and PDG for each block of phase A and B (dominant side). Trajectories are obtained from the projection of 3D curves in the 2D coronal plane. The thickness of lines represents the dispersion of data around the mean trajectory.

In Figure 3, the averaged dominant hand trajectories are shown for both phase A and B, considering each block separately, highlighting differences especially in grasping and moving the more proximal blocks (from number 5 to number 10). The kinematic parameters calculated from the hand trajectories are depicted in Table 3; statistically significant inter-group differences have been found in all parameters. The mean velocity and the mean acceleration showed significantly lower values in PDG than CG. The DLJ index revealed that subjects with PD had lower movement smoothness than the able-bodied ones.

## 4. Discussion

This observational pilot study was conducted on 11 subjects with PD compared to 11 able-bodied subjects in order to assess the upper body kinematics during the targeted BBT with an ecological IMU-based system. To this extent, the IMU-based targeted BBT was analyzed for both the dominant and non-dominant upper limbs. All subjects were able to easily perform the requested motor tasks.

The analysis of the IMU data allowed us to calculate the joint angle kinematics. The outcomes from the able-bodied subjects were in accordance with the literature on similar studies based on stereophotogrammetry [16,17]. The results from subjects with PD allowed us to characterize the quality of the movements and the compensatory strategies typical of this disease [13,30,31]. Specifically, the wrist evidenced a significant higher mean flexion and ulnar deviation in the PDG compared to the CG, while the wrist F-E mean angular trajectories (Figure 2) were similar in both groups. The ROEs were significantly higher in the PDG than the CG in all wrist angles except for the URD dominant arm, Phase A. The forearm registered significantly higher supination values in the CG compared to the PDG; the variations of this angle over time were similar during the trial execution. The shoulder depicts significant lower ROEs in PDG than CG in F-E, A-A and R angles. Moreover, the mean shoulder F-E trajectories of the two groups were similar from 0% to 50% of trial completion, while when the block was carried over the partition the PDG evidenced a reduced shoulder flexion. This outcome was found in both dominant and non-dominant arms and in both phases A and B. The shoulder A-A is characterized by a significant smaller ROE in all motor tasks, thus revealing a limited angular excursion in PDG. Conversely, the PDG significantly rotated the shoulder more than the CG, showing significant inter-group differences in the mean angle and the ROE. Therefore, we can affirm that the PDG partially involved the shoulder during the execution of the motor tasks, except for the shoulder R, which seems to compensate for the limited ROE in FE and AA. The trunk exhibited higher ROEs in PDG than CG, thus confirming the employment of a compensatory strategy in subjects with PD [13,30,31].

The qualitative analysis of the hand trajectories (Figure 3) showed that the subjects with PD moved the end-effector like the able-bodied subjects in the movement of the first blocks (grasp and release of blocks 1–4), while they tended to decrease the range of motion and the precision in the subsequently blocks (grasp and release of blocks 5–10). Moreover, the PDG executed the movement with a significant lower mean velocity and mean acceleration of the hand in all considered motor tasks. The DLJ shows that the subjects with PD moved the end-effector with lower smoothness, in accordance with the literature on the quantitative analysis of bradykinesia and rigidity in PD [29,31].

The results of this study evidence that the proposed IMU-based targeted BBT is able to quantitatively and easily assess upper body kinematics during a test of manual dexterity. Moreover, the analysis of joint angle trajectories allows to characterize movements’ quality and to find the compensatory strategies of subjects with PD. The analysis of such compensatory motor approaches could help the understanding of functional gain in a perspective of personalized punctual evaluation of patients with PD undergoing rehabilitative treatments.

The proposed IMU-based targeted BBT protocol is feasible, easy-to-do, low-cost and ecological. All recruited subjects participated in the experiments and executed the motor tasks without any difficulty. In a period in which motor rehabilitation increasingly needs an objectification of motor performance to personalize treatment, this system allows performing a quantitative movement analysis easily and accurately in the clinical setting.

The main limitations of the study are the restricted number of recruited subjects and the inclusion of PD subjects with a moderate impairment only. Future studies should consider a higher sample size to confirm our outcomes. Moreover, the analysis of subjects with different pathologies and motor impairment would allow us to discriminate different motor strategies.

## 5. Conclusions

An IMU-based targeted BBT allowed to analyze the upper body kinematics in subjects with PD and able-bodied persons. The analysis of joint angles and hand trajectories characterized the quality of the movements in the two groups and evidenced the compensatory strategies of subjects with PD. The obtained results suggest future studies on different pathologies since the IMU-based BBT could be a potential system for the standardized assessment of the upper limb in the clinical setting.

## Figures and Tables

**Table 1:** Clinical and demographic characteristics of the enrolled subjects.

	CG (N = 11)			PDG (N = 11)		
**Age (years)**	66.90	±	5.80	72.00	±	8.20
**Gender Male, n (%)**	6 (54.5%)			6 (54.5%)		
**BBT—dominant side (n° cubes)**	66.90	±	10.25	56.73	±	12.97
**BBT—non dominant side (n° cubes)**	64.18	±	8.46	52.36	±	11.27
**Affected side dx, n (%)**	-			4 (36.3%)		
**Hoehn&Yahr**	-			2.5 (2–3)		
**UPDRS I**	-			5 (0–8)		
**UPDRS II**	-			19 (13–22)		
**UPDRS III**	-			20 (18–32)		
**UPDRS VI**	-			5 (0–10)		
**UPDRS TOT**	-			51 (38–67)		
**TCT**	-			61 (42–87)		

*Abbreviations: BBT, Box and Blocks Test; UPDRS, Unified Parkinson’s Disease Rating Scale; TCT, Trunk Control Test; CG, Control Group; PDG, Parkinson’s Disease Group. Notes: Data are reported as mean ± standard deviation or frequency with percentage (%) or median (min–max).*

**Table 2:** Joint angles parameters calculated for each phase (A and B) of the targeted BBT tasks.

Joint Angles																										
			Dominant Arm												Non-Dominant Arm											
			Phase A						Phase B						Phase A						Phase B					
Joint		Group	Mean Angle			ROE			Mean Angle			ROE			Mean Angle			ROE			Mean Angle			ROE		
Wrist	F-E	CG	**7.7**	**±**	**17.5**	**19.4**	**±**	**8.3**	**8.7**	**±**	**18.2**	**16.1**	**±**	**6.4**	**14.2**	**±**	**13.7**	**18.8**	**±**	**8.9**	**11.9**	**±**	**13.2**	**15.5**	**±**	**7.2**
		PDG	**22.4**	**±**	**23.6**	**26.9**	**±**	**12.5**	**24.5**	**±**	**23.5**	**22.3**	**±**	**13.8**	**18.8**	**±**	**14.4**	**21.5**	**±**	**7.6**	**22.7**	**±**	**14.9**	**17.6**	**±**	**7.8**
	URD	CG	**6.0**	**±**	**9.6**	20.3	±	8.4	**−1.9**	**±**	**9.8**	**11.6**	**±**	**6.4**	**2.2**	**±**	**13.5**	**23.9**	**±**	**10.6**	**−4.7**	**±**	**13.9**	**17.8**	**±**	**11.1**
		PDG	**9.4**	**±**	**18.7**	20.7	±	11.8	**7.3**	**±**	**20.4**	**18.8**	**±**	**13.0**	**10.7**	**±**	**19.7**	**16.5**	**±**	**6.7**	**6.8**	**±**	**20.7**	**14.3**	**±**	**10.3**
Forearm	P-S	CG	**105.1**	**±**	**22.6**	13.9	±	9.0	**108.7**	**±**	**23.2**	**11.5**	**±**	**6.7**	**104.8**	**±**	**12.9**	11.7	±	3.8	**106.8**	**±**	**14.3**	12.0	±	3.7
		PDG	**92.8**	**±**	**15.2**	19.9	±	16.1	**95.7**	**±**	**14.8**	**18.6**	**±**	**12.5**	**97.8**	**±**	**16.5**	13.6	±	9.2	**100.9**	**±**	**16.3**	13.5	±	7.8
Elbow	F-E	CG	85.6	±	24.2	17.7	±	7.0	87.7	±	26.1	19.4	±	7.8	76.5	±	14.4	19.0	±	8.2	77.4	±	15.4	19.8	±	9.1
		PDG	89.5	±	41.5	17.6	±	7.7	90.9	±	41.6	19.5	±	8.6	79.3	±	27.0	25.3	±	46.6	74.8	±	44.0	38.4	±	76.3
Shoulder	F-E	CG	29.5	±	12.4	**35.7**	**±**	**11.5**	26.5	±	13.4	**38.5**	**±**	**12.0**	**29.0**	**±**	**10.3**	**38.2**	**±**	**9.7**	**27.3**	**±**	**10.1**	**39.7**	**±**	**9.9**
		PDG	27.2	±	27.8	**26.2**	**±**	**11.4**	24.3	±	27.3	**28.3**	**±**	**13.2**	**42.5**	**±**	**28.5**	**34.8**	**±**	**12.0**	**38.8**	**±**	**33.3**	**34.7**	**±**	**12.8**
	A-A	CG	40.3	±	14.3	**25.6**	**±**	**7.0**	44.4	±	14.2	**29.3**	**±**	**8.0**	37.1	±	10.2	**26.4**	**±**	**7.9**	39.5	±	10.2	**29.8**	**±**	**7.6**
		PDG	39.1	±	20.3	**21.2**	**±**	**8.5**	41.2	±	18.5	**25.2**	**±**	**9.5**	36.1	±	14.6	**21.7**	**±**	**12.9**	37.4	±	14.2	**26.7**	**±**	**14.1**
	R	CG	**−39.8**	**±**	**13.1**	**38.6**	**±**	**12.2**	**−44.0**	**±**	**15.3**	**41.1**	**±**	**12.3**	**−39.9**	**±**	**10.0**	**40.9**	**±**	**10.1**	−41.8	±	10.9	**41.9**	**±**	**9.2**
		PDG	**−52.8**	**±**	**32.0**	**28.5**	**±**	**12.3**	**−56.5**	**±**	**30.9**	**31.2**	**±**	**11.8**	**−34.9**	**±**	**20.8**	**26.8**	**±**	**9.3**	−41.0	±	26.0	**28.7**	**±**	**11.2**
Trunk	F-E	CG	10.8	±	7.9	**2.8**	**±**	**1.6**	12.0	±	7.8	**2.5**	**±**	**1.7**	**8.6**	**±**	**4.3**	**3.0**	**±**	**1.4**	**9.6**	**±**	**4.3**	3.0	±	1.6
		PDG	11.7	±	6.1	**4.3**	**±**	**2.3**	11.7	±	6.6	**4.6**	**±**	**3.7**	**15.2**	**±**	**8.1**	**4.0**	**±**	**2.3**	**15.6**	**±**	**8.1**	3.4	±	2.2
	R	CG	**3.0**	**±**	**6.6**	6.9	±	3.8	**3.5**	**±**	**7.3**	**7.4**	**±**	**3.5**	**−10.5**	**±**	**9.0**	8.0	±	3.1	**−11.9**	**±**	**10.8**	8.6	±	2.9
		PDG	**6.0**	**±**	**19.0**	8.1	±	4.3	**5.7**	**±**	**19.8**	**9.8**	**±**	**4.8**	**−5.9**	**±**	**10.2**	7.4	±	3.9	**−6.2**	**±**	**13.1**	8.4	±	3.6

*Abbreviations: F-E, Flexion-Extension; URD, Ulnar Radial Deviation; P-S, Prone-Supination; A-A, Abduction-Adduction; R, Rotation. CG, Control Group; PDG, Parkinson’s Disease Group. Notes: Data are reported as mean ± standard deviation. The data marked in bold denotes significant inter-group difference (p < 0.05).*

**Table 3:** Averaged hand trajectory parameters calculated from the dominant and non-dominant arms in phases A and B.

Hand Trajectory Parameters													
Parameter	Group	Dominant						Non-Dominant					
		Phase A			Phase B			Phase A			Phase B		
V_m_ (cm/s)	CG	**40.45**	**±**	**2.66**	**42.13**	**±**	**2.86**	**41.11**	**±**	**2.78**	**38.47**	**±**	**2.69**
	PDG	**31.79**	**±**	**3.36**	**36.33**	**±**	**3.73**	**37.22**	**±**	**3.41**	**35.69**	**±**	**3.50**
A_m_ (cm/s^2^)	CG	**687.45**	**±**	**59.05**	**695.96**	**±**	**65.33**	**665.95**	**±**	**51.92**	**602.55**	**±**	**48.88**
	PDG	**570.18**	**±**	**65.98**	**622.77**	**±**	**79.83**	**618.15**	**±**	**63.82**	**548.90**	**±**	**54.95**
DLJ	CG	**−2.95**	**±**	**0.78**	**−2.40**	**±**	**0.49**	**−2.63**	**±**	**0.69**	**−2.97**	**±**	**0.69**
	PDG	**−8.85**	**±**	**6.54**	**−7.21**	**±**	**4.78**	**−4.91**	**±**	**1.48**	**−4.56**	**±**	**1.59**

*Abbreviations: V_m_, mean velocity; A_m_, mean acceleration; DLJ, DimensionLess Jerk index; CG, Control Group; PDG, Parkinson’s Disease Group. Notes: Data are reported as mean ± standard deviation. The data marked in bold denote significant inter-group difference (p < 0.05).*

## Data Availability

The data presented in this study are available on request from the corresponding author.
